# Factors and Mechanisms Affecting Arsenic Migration in Cultivated Soils Irrigated with Contained Arsenic Brackish Groundwater

**DOI:** 10.3390/microorganisms12122385

**Published:** 2024-11-21

**Authors:** Wenjing Dai, Rongguang Shi, Xiaodong Li, Zhiqi Zhao, Zihan Xia, Dongli Li, Yan Li, Gaoyang Cui, Shiyuan Ding

**Affiliations:** 1 School of Earth System Science, Tianjin University, Tianjin 300072, China; 2 School of Earth Science and Resource, Chang’an University, Xi’an 710054, China; 3 Agri-Environmental Protection Institute, Ministry of Agriculture and Rural Affairs, Tianjin 300072, China; 4 College of Geography and Environmental Science, Henan University, Kaifeng 475004, China

**Keywords:** arsenic, migration mechanism, iron oxides, brackish groundwater, cultivated soil, genetic sequences

## Abstract

Contained arsenic (As) and unsafe brackish groundwater irrigation can lead to serious As pollution and increase the ecological risk in cultivated soils. However, little is known about how Fe oxides and microbes affect As migration during soil irrigation processes involving arsenic-contaminated brackish groundwater. In this study, the samples (porewater and soil) were collected through the dynamic soil column experiments to explore the As migration process and its effect factors during soil irrigation. The results showed that the As concentration in porewater samples from the topsoil was enriched compared to that in the subsoil, and the main solid As fractions were strongly adsorbed or bound to amorphous and crystalline Fe oxides. The aqueous As concentration and the solid As fractions indicated that reductive dissolution and desorption from amorphous Fe oxides were the primary mechanisms of As release at the topsoil and subsoil, respectively. Meanwhile, *Sphingomonas*_sp., *Microvirga_ossetica* and *Acidobacteriota_bacterium* were the dominant microbes affecting As biotransformation by arsenate reductase gene (*arsC*) expression. Accompanied by the Eh and competitive ions concentration change, amorphous Fe oxide dissolution increased to facilitate the As release, and the changes in the microbial community structure related to As reduction may have enhanced As mobilization in soils irrigated by As-containing brackish groundwater.

## 1. Introduction

Due to agricultural and industrial activities, arsenic (As) contamination in soils has gradually become a serious problem [1]. In the arid and semi-arid regions of northern China, the use of brackish groundwater contaminated with As has been widely recommended for agricultural irrigation, particularly during the dry season to alleviate freshwater shortages [2]. However, the use of As-contaminated groundwater for agricultural irrigation leads to the accumulation of As in the crop root zone, which far exceeds the background and safe levels for agricultural soils [3,4,5]. It has been demonstrated that there is a significant correlation between As concentrations in irrigation water or agricultural soils and the As accumulated in crops [6,7]. Additionally, the leaching process affects the biogeochemical cycle of redox-sensitive elements [8,9], which may influence the distribution and migration of As in the soil [10,11,12]. Therefore, it is essential to understand the mechanisms of As migration and the factors affecting it during soil irrigation with As-contaminated brackish groundwater to assess the ecological risks for cultivated soils during the irrigation process.

Fe oxides, due to their redox reactivity and surface-specific adsorption capacities, are considered to be important sources and sinks for As in soils [13,14]. The fixation and release of As are associated with Fe oxides [15,16,17], and this process usually involves the transformation of Fe minerals or other forms that retain As [18,19]. Previous studies have focused on how Fe oxides affect As mobilization in paddy and wetland soils, which experience significant redox changes, while the importance of the irrigation process in dryland soils has been largely overlooked [20,21,22]. The chemical stability of Fe oxides is key to As migration, as their interactions, along with the precipitation and dissolution of these oxides and their transformation into secondary minerals, directly affect the immobilization and mobilization of As [23]. In anoxic conditions, As is released by the reductive dissolution of Fe oxides; however, weakly crystalline Fe oxides combined with Fe^2+^ may recrystallize into other crystalline Fe oxides, which immobilize the released As in soils [18,24]. When transitioning to aerobic conditions, O_2_ diffusion leads to the oxidation of Fe^2+^, forming new weakly crystalline precipitates that facilitate the isolation of As from the soil solution [25]. It remains unclear how different Fe oxides influence the As biogeochemical cycle in cultivated soils during the irrigation process.

Although brackish groundwater irrigation provides a solution to agricultural water needs in arid regions, it may lead to elevated salinity and affect the fate and bioavailability of As in soils [26,27,28]. Experimental studies that accurately identify the biogeochemical processes and mechanisms of As under dynamic redox conditions in dryland cultivated soils were still scarce. The moisture and solutes in irrigation water can alter the properties of Fe oxides and the community structure and function of soil microorganisms, which affects As release from soils to porewater. Nitrate oxidizes Fe^2+^ through chemical and biological reactions to produce Fe oxides enhancing As immobilization [16,29,30]. Phosphates form inner-sphere complexes with the surface of Fe oxides, which leads to a weakening of the adsorption capacity for As by competing with the adsorption sites on the surface of Fe oxides due to their structural similarity to As(V) [31,32,33]. In addition, the presence of anions (such as silicates and sulfates) also affects the stability of Fe-As complexes [34]. Previous studies have focused more on As biogeochemical processes in paddy soils under freshwater irrigation; few studies have explored the mechanisms of As migration by soil microbes in dryland soils under alternating dry and wet conditions, especially regarding their effects on microorganisms with As reduction and oxidation functions [12,35,36]. The infiltration of brackish water affects the soil mineral fractions, the microbial community composition, and the leaching of heavy metals [37,38,39]. It is currently unclear whether the environmental behavior of As in soils will change over time due to dynamic redox conditions and the effects of brackish water irrigation.

Therefore, a dynamic soil column experiment was conducted to simulate the As migration process and distribution in dryland cultivated soils after irrigation with As-contaminated brackish groundwater irrigation in this study. By setting different sequential extraction procedures, the change in the content for solid-phase As fractions throughout the soil profile provided reliable information for exploring the As migration mechanism. The objectives of this study were as follows: (1) to investigate the spatial and temporal distribution of As in liquid and solid phases bound to Fe oxides (amorphous and crystalline) during the irrigation process; (2) to investigate the As migration mechanism during this process; (3) to quantitatively evaluate the effect of factors of brackish groundwater irrigation on As migration. Overall, the objectives of the study were to determine the controlling mechanisms and influencing factors for As migration during the irrigation process using As-contaminated brackish groundwater in dryland soils. The findings of this study contribute to a better understanding of As migration during brackish groundwater irrigation, and they provide significant guidance for the prevention and control of As pollution in agricultural soils for high As background areas.

## 2. Materials and Methods

### 2.1. Column Experiments

**Figure 1:**
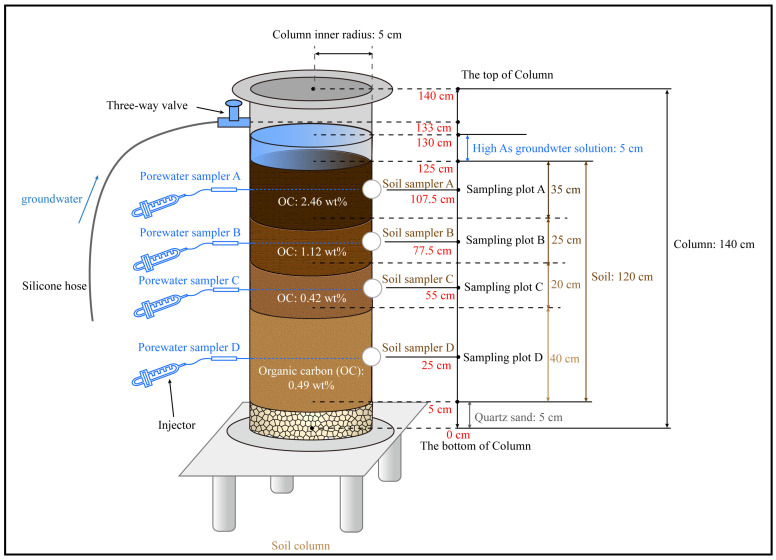
Schematic diagram of the soil column experiment set-up.

The experimental setup for the column experiments consisted mainly of acrylic glass tubes with an inner diameter of 10 cm and a height of 14 cm (Figure 1). Ventilation valves were provided at the top and bottom of the column and were closed during the experiment. A water inlet was located on the left wall of the column 5 cm from the top and was flanked by four evenly spaced porewater and soil sample ports on each side, arranged in parallel. The water inlet and the porewater sampling ports were fitted with plastic screw caps and sealed with a gasket to ensure a leak-proof connection between the column and the screw caps. The soil sampling ports were opened when the soil samples were collected but remained closed during the experiment. In addition, a peristaltic pump and a 7 L reservoir were connected to the water inlet through a hose. Irrigation water was stored in the reservoir and pumped by the peristaltic pump into the soil column at 60 rpm/min, with the water flowing in a downward direction. During the irrigation period (flooded condition), the irrigation water level was maintained at 3 to 5 cm above the soil surface. The experiment concluded the irrigation when the water interface reached the bottom of the soil column, which was the beginning of the reaction. No further irrigation was carried out during the 35-day reaction cycle. Porewater and soil samples were collected on the 1st, 3rd, 7th, 15th, 25th, and 35th days of the leaching experiment, respectively. The column experiment was not replicated. 

In the column experiments, brackish groundwater containing As collected from the northern part of Hangjin Houqi in the Hetao basin was selected as the simulated irrigation water, with an initial As concentration of 8.0 μg/L. The water was aerated with N_2_ for 30 min to remove the dissolved oxygen. Agricultural soils were selected as typical dryland arable soils in the Hetao irrigation area, with maize as the main planting crop. The arable soil was collected in layers of 0~35 cm, 35~60 cm, 60~80 cm, and 80~120 cm. The stratification was based on the carbon content and mineral composition of the soil in different layers. Afterward, impurities (such as branches, stones, and roots) were removed, and the soil was air-dried and sieved through a 10-mesh sieve. The soil was then evenly mixed and filled into the columns, maintaining the same order as the field sampling horizons. Quartz sand (0.25 μm) was also placed at the bottom to avoid soil loss. Porewater and soil samples collection sites were located in the middle of each soil layer to avoid unevenness in the collection of samples from each layer. The physicochemical indicators associated with the soil samples from each layer are detailed in Table S1.

### 2.2. Sample Analytical Methods

#### 2.2.1. Aqueous Phase Analysis

A total of 24 porewater samples were collected during the experiment. The aqueous phase samples were all collected from a soil porewater sampler (Rhizo Core Solution Sampler 19.21.24F, Rhizo, Amsterdam, The Netherlands) in each layer. Approximately 40 mL of aqueous phase samples for analysis were filtered using a syringe-driven 0.45 μm filter and collected in acid-washed centrifuge tubes (50 mL). HCO_3_^−^ concentration was determined by HCl titration. Anions and cations concentrations were determined using a dual-system ion chromatograph (ICS 5000+, Thermo Fisher, Austin, TX, USA). PO_4_^3−^ concentrations were analyzed using a nutrient salts analytical instrument (San++, Skalar, Breda, The Netherlands). Total As, Fe, Mn, and Mo concentrations were determined by ICP–MS (ICP–MS 8900, Agilent, Santa Clara, CA, USA), expressed by “L” (liquid). All samples to be detected were stored in a refrigerator at 4 °C, detected within 15 days, and were analyzed by the Institute of Surface—Earth System Science, Tianjin University, Tianjin, China.

#### 2.2.2. Solid Phase Analysis

A total of 24 soil samples were collected during the column experiments. The solid samples were collected from soil sampling sites established on the flanks of each column layer. The method described by Keon et al. [40] and Poulton [41] was followed with some modifications. The steps of sequential extraction are shown in Table S2. Briefly, the collected wet soil samples (with a dry weight of about 0.6~0.8 g) were directly placed in 50 mL centrifuge tubes, and the sequential extraction of As and Fe was carried out at a solid (wet weight) liquid ratio of 1:30. Before the sequential extraction was initiated, the soil samples were washed once with Milli-Q water (Merck Milli-Q, St. Quentin Fallavier, France) to remove free water from the soil. Fresh extractants were prepared by dissolving the chemicals in deoxygenated Milli-Q water (N_2_ degassed) at room temperature. A total of 30 mL of extractant was added at each step. Sealed centrifuge tubes containing the extractant and samples were added and were subjected to constant shaking in a thermostatic oscillator at 180 rpm/min for 25 °C. At the end of each extraction step, the suspension was centrifuged in a centrifuge at 4000 rpm/min. The solid, after centrifugation in the previous step, was washed with Milli-Q water after 30 min of shaking and was used for the next extraction. The supernatant was filtered using a syringe-driven 0.45 μm filter. The As, Fe, and Mn contents of the extracted samples were analyzed by ICP–MS, expressed by “S” (solid). Blanks, extraction standards (European Commission, BCR–701, Europe), and samples were prepared in parallel using the same method as that for the sample extraction procedure. The recoveries of both sequential extraction methods ranged from 80 to 120%.

#### 2.2.3. Illumina Novaseq Sequencing and Quality

Metagenome sequencing was performed using the Illumina Novaseq high-throughput sequencing platform (San Diego, IL, USA) to obtain raw data on microorganisms in soil samples. A total of approximately 8 million sequences were obtained for each soil sample. The software Fastp (v0.20.0) was utilized to remove splice sequences, retaining only high-quality reads. After that, short sequences were spliced and assembled using Megahit (v1.1.2). A total of 10,267,799 contigs were obtained with N50 at 522 bp. Gene prediction was performed on the contigs resulting from the splicing process using Prodigal (v2.6.3). Non-redundant gene sets were constructed using CD-HIT (v4.6.1). The number of genes in all samples before de-redundancy was 2,490,396, while the number of genes in the non-redundant gene set was 1,655,333. The non-redundant gene set was constructed with gene sequence clustering similarity (identity) ≥ 0.9 and gene sequence clustering coverage (coverage) ≥ 0.9. The gene sequences from the splicing results were predicted using the SOAPaligner soap2.21 release software to build the gene set for As.

### 2.3. Data Processing and Analysis

Experimental data were analyzed using Origin 2024 and R programming language. The Spearman correlation coefficient between different environmental variables and significant differences were both analyzed using R 4.1.1and Origin 2024. The saturation index (SI) was calculated using the PHREEQC version 3.7.3 software. Redundancy analysis (RDA) was performed using Canoco 5 software. The random forest model was implemented with the R package “randomForest” and “rfpermute” to identify the most important environmental factors. The partial least squares path modeling (PLS-PM) was established by the R package “plspm” to calculate the loadings (or weights), path coefficients (r), and the goodness of fit. These parameters were used to evaluate the direct and indirect effects of observed variables on each latent variable.

## 3. Results and Discussion

### 3.1. The Vertical and Temporal Changes of Soil Indexes After Leaching

#### 3.1.1. Chemical and Biological Characteristics for Soil Porewater Samples

**Figure 2:**
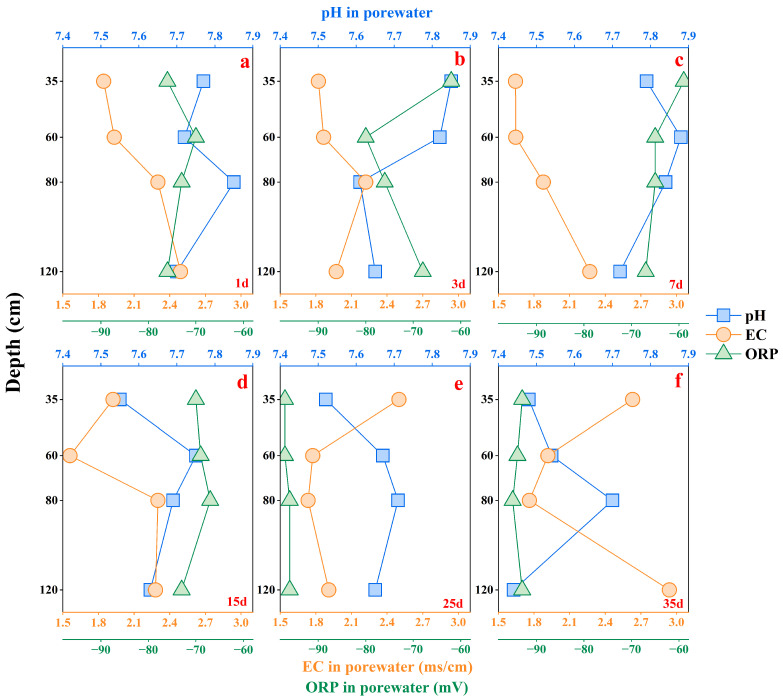
Physical and chemical parameters (pH, EC, and ORP) of soil porewater after the leaching experiment. (**a**–**f**) represent the measured pH, EC, and ORP at 1, 3, 7, 15, 25, and 35 days, respectively.

The chemical characteristics of 24 soil porewater samples collected after a 35-day cycle of soil column leaching experiments are listed in Table S3. As shown in Figure 2, the porewater was neutral or weakly alkaline, with pH values ranging from 7.44 to 7.88, which exhibited minimal variation with soil depth and time. The ORP values of the porewater ranged from −97 mV to −59 mV, indicating that the soil was a weakly reducing environment during the high As groundwater leaching process. Moreover, Fe^2+^ was below the detection limit in some porewater samples, while NO_2_^−^ was detected in all samples. As the pore space in the column was filled with the experimental water, other diffusion in the air within the column may be limited. Therefore, ORP fluctuated during the first 7 days of the leaching process. The EC values of the porewater ranged from 1.56 to 2.94 ms/cm, and the main cations and anions were Na^+^, Ca^2+^, and HCO_3_^−^, SO_4_^2−^, respectively. Ca^2+^ originated from the dissolution of carbonate and gypsum (Figure S1a,b). HCO_3_^−^ came mainly from carbonate mineral dissolution and microbial respiration in the soil (Figure S1c,d). In the early stages of leaching, the salts in the porewater primarily came from irrigation water. As the leaching process continued, the salts accumulated in the soil began to dissolve. High salt loading may affect the mobilization of dissolved As by controlling the adsorption and desorption processes of As on Fe oxides [42,43,44]. 

#### 3.1.2. The Total As, Fe, and Mn Concentration for Soil Porewater Samples

**Figure 3:**
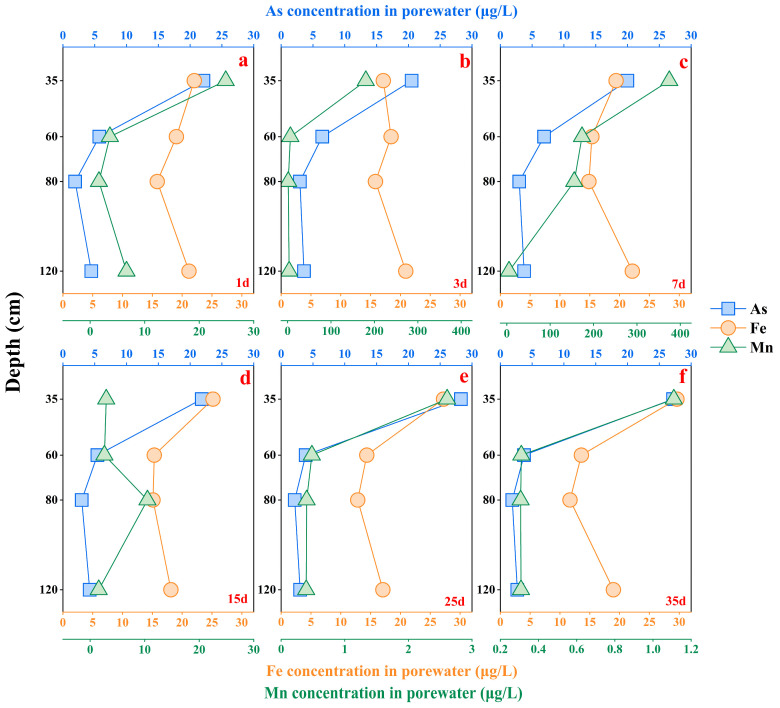
The concentration of As, Fe, and Mn in soil porewater in different reaction times. (**a**–**f**) represent the measured Total As, Fe and Mn concentration at 1, 3, 7, 15, 25, and 35 days, respectively.

As shown in Figure 3, the total As concentration of the porewater samples ranged from 1.88 to 28.3 μg/L, with a mean value of 8.68 μg/L. Among the 24 porewater samples collected, those from layer #A were significantly enriched in As, and all the As concentrations exceeded the safety limit of 10 μg/L. The risk of As contamination in layer #A was critical to human health because it is located in a crop cultivation area. the As concentrations in the other layers (layers #B, #C, and #D) were below 10 μg/L, with the lowest concentration being 2.47 μg/L in the porewater sample from layer #C. The As in the porewater originated from the experimental water and the leaching of soil As, which migrated vertically downward with the water flow. The correlation analysis revealed that the As concentration had a significant positive correlation with the concentrations of Fe and Mn (Figure S2). Moreover, we can clearly see from the correlation between ORP and the total As and Fe concentrations that, in layer #A, ORP has a significant negative correlation with total As and total Fe concentrations (Figure S3). This suggests that the decrease in ORP in layer #A may lead to an increase in total As and Fe concentrations in porewater. It is possibly related to the reductive dissolution of Fe/Mn oxides in the soil. Genome sequencing results (Figure S4) indicate that functional microorganisms associated with As are mainly dominated by *s_Acidobacteriota_bacterium* and *s_Chloroflexota_bacterium* with a percentage of 12.5% and 10.5%, respectively.

#### 3.1.3. The As Content Based on Different Fractions for Soil Samples

**Figure 4:**
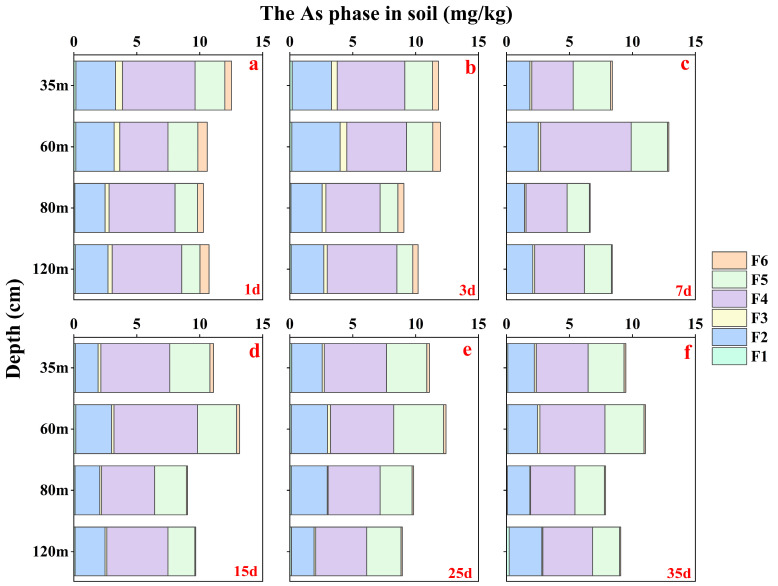
The As content for soil samples based on different extraction phases. (**a**–**f**) represent the measured the As content of different fraction at 1, 3, 7, 15, 25, and 35 days, respectively.

In order to determine the potential influence of soil Fe oxides on soil As migration, the present study was conducted to geochemically analyze solid-phase As in various forms using different extractants and the As content and standard errors with each solid phase As of extraction standard (BCR-701) are shown on Figure S5. The results (Figure 4) indicate that solid-phase As comprises a minimal amount of non-specifically adsorbed As (F1). As co-precipitated with carbonate (F3) and Magnetite (F6) was observed in only small amounts. Most of the solid phase As in soil was primarily present as specifically adsorbed As (F2), As bound to amorphous Fe oxides (F4), and crystalline Fe oxides (F5). These three forms of solid-phase As (F2, F4, and F5) accounted for over 80% of the total soil As content. There was no clear vertical distribution of the content of different forms of solid-phase As in the soil, slight differences were observed between the layers. In layer #A, solid As was more abundant as bound to amorphous Fe oxides and gradually decreased with soil depth. And other layers were dominated by F2. With increased leaching time, the solid-phase As content of both F2 and F4 in the soil decreased compared to pre-leaching levels, F5 increased significantly. It indicates that solid-phase As bound to crystalline Fe oxides is more stable during soil leaching in a weakly reduced soil environment. 

### 3.2. The Sources of As for Soil Porewater

**Figure 5:**
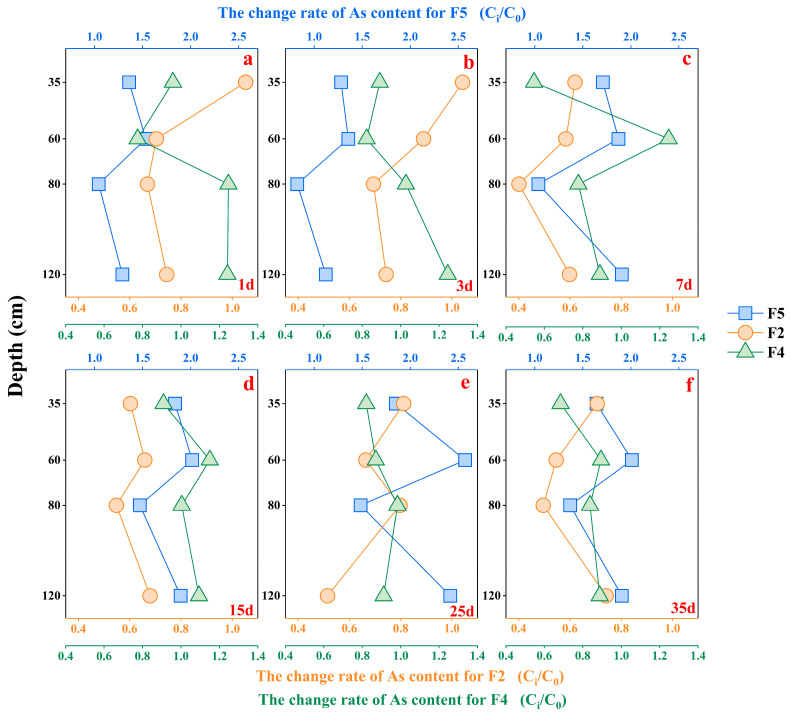
The change trend of the adsorbed As (F2), amorphous Fe oxides combined As (F4), and crystallized Fe oxides combined As (F5) contents with time for soil samples. (**a**–**f**) represent the measured the change rate of As content for F2, F4, and F5 at 1, 3, 7, 15, 25, and 35 days, respectively.

In this study, specifically adsorbed As was identified as one of the main forms of solid-phase As occurrence. Compared with the soil before leaching (0d), the content of adsorbed As decreased at different layers after leaching (the change rate < 1.0, Figure 5). The results indicate that the soil leaching process led to the desorption of As, although the content of As released through desorption varied across different layers. Meanwhile, a significant positive correlation was observed between the As concentration in porewater and strongly adsorbed As in samples with As concentrations < 10 μg/L (layers #B, #C, and #D) (Figure S6). It suggests that the desorption process was the main mechanism for As release in layers #B, #C, and #D during the leaching process. However, there was no significant relationship between the As concentration in porewater and the adsorbed As (F2) (layer #A) where the As concentration was greater than 10 μg/L (Figure S6). In addition, the contents of As desorbed from pore water samples with As concentrations lower than 10 μg/L were higher than that from samples with concentrations over 10 μg/L. This indicates that there are other release mechanisms in layer #A aside from the desorption process, such as the dissolution of As-loaded Fe oxides. 

The As contained in amorphous Fe oxides (F4) was dissolved in layer #A during the soil leaching process (the change rate < 1.0, Figure 5). Moreover, a significant positive correlation between As concentration and Fe^2+^ concentration suggests that the reductive dissolution of Fe oxides was another cause of As release in layer #A. As the leaching process progressed, the As-containing Fe oxides were gradually dissolved as the reducing environment intensified. This led to a gradual increase in both As and Fe concentrations while the solid-phase As content decreased. Furthermore, the migration of As was significantly influenced by the Fe fraction in the soil. Amorphous Fe oxides exhibit a relatively high adsorption affinity for As compared to other crystalline Fe oxides. Due to their inherent instability stemming from low crystallinity, prolonged immersion in reducing water and soil environments may lead to the reductive dissolution of amorphous Fe oxides, resulting in the release of As. In addition to being affected by redox changes in soil, the reductive dissolution of amorphous Fe oxides may also be related to soil microbial activity [45,46]. Several studies have confirmed that heterotrophic Fe-reducing bacteria contribute to the dissolution of Fe oxides, transforming Fe oxides with high surface area and low crystallinity into secondary minerals with low surface area while promoting the release of As [46,47,48,49]. Therefore, the As migration process is closely linked to Fe oxides in the soil.

### 3.3. The Effect Factors for As Migration in Cultivated Soils

#### 3.3.1. The Effect of Fe Oxides for As Migration

In this study, the desorption process facilitated the release of As from Fe oxides in soil. Previous studies have indicated that Fe oxides and the form of dissolved As significantly affected their behaviors in soil [50,51,52,53]. Figure S7 shows the As in the porewater was mainly in the form of HAsO_4_^2−^ and H_3_AsO_3_. The dissolved As formed surface complexes with Fe oxides in both the inner and outer spheres, which were connected by monodentate and bidentate ligands [31,54,55]. In this study, the adsorbed As at layer #A was mainly assigned to crystalline Fe oxides (Figure S8a), whereas layers #B, #C, and #D were mainly loaded on amorphous Fe oxides (Figure S8b). Additionally, the As content of the combined amorphous (F4) and crystalline Fe oxides (F5) exhibited a different trend over leaching time (Figure 5 and Figure S9). The amorphous Fe oxides were in a metastable state; the As-loaded amorphous Fe oxides would transform and grow to thermodynamically more stable crystalline Fe oxides over time [34,56]. During this process, the specific surface area of Fe oxides and adsorption sites gradually decreases on the surface [18,57]. Previous studies suggested As adsorbed in ferrihydrite was continuously released during the transformation of Fe oxides [58,59,60]. This release resulted from the encapsulation of mineral micropores, reduction of specific surface area, and lack of surface site density during the crystalline transformation and growth of ferrihydrite [18,61,62]. Therefore, when the adsorption sites on the surface of the Fe oxides disappear or are incapacitated to adsorb As during the conversion of amorphous to crystalline Fe oxides, this may provoke the desorption of As from the surface of iron oxides [63]. Furthermore, it can also be seen from the positive relationship between the total As concentration in porewater and the As content in crystalline (Figure S10). If As was not desorbed during the Fe oxide crystallization process, it could be introduced into the crystalline structure of the crystalline Fe oxide and remain stably immobilized in the lattice of transformation products of Fe oxide [19,64,65]. Moreover, Fe-As complexes on the surface of amorphous Fe oxides also adsorbed Fe^3+^ in porewater due to hydrolysis and dissolution processes. Consequently, the dissolved As can be adsorbed again by surface complexation, and finally form surface precipitation [66]. Thus, the As migration was influenced by Fe oxides in soil. 

#### 3.3.2. The Effect of Inorganic Ions for As Migration

**Figure 6:**
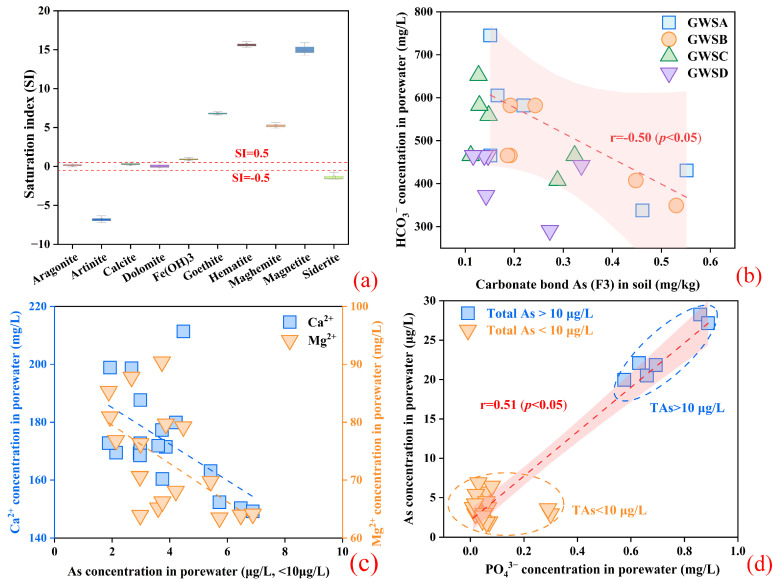
(**a**) Saturation index of carbonate minerals and Fe oxide minerals; (**b**) Relation between the contents of As combined with carbonate and HCO_3_^−^ concentration; (**c**) Relation between the As and Ca^2+^, Mg^2+^ concentration; (**d**) Relation between the As concentration and PO_4_^3−^ for porewater samples.

Whereas the presence of competing different ions in the soil solution also influences the adsorption of As by Fe oxides [67,68,69]. HCO_3_^−^ is one of the major anions in porewater samples. The extent of the competitive effect of HCO_3_^−^ on the adsorption of low levels of arsenate by Fe oxides is still controversial at near-neutral pH, especially in the presence of stronger competing ions such as PO_4_^3−^. Mai et al. (2014) examined the effect of 1 to 10 mM HCO_3_^−^ on the As adsorption onto aquifer sediments at the Nam du, and showed that the presence of HCO_3_^−^ did not affect As complexation on the sediment surface [70]. However, it was discovered that HCO_3_^−^ displaced As(III) from sediment surface complexes, leading to the movement of As [71]. If the mechanism of As release were simply desorption due to HCO_3_^−^ substitution, this process should take place in soil layer #A, which already had a high HCO_3_^−^ concentration. However, As release was clearly not dominated by desorption in layer #A. In some dynamic adsorption experiments with synthetic Fe minerals, the combination of HCO_3_^−^ and Ca^2+^, Mg^2+^ led to the desorption of As from ferrihydrite due to the saturation of the ferrihydrite surface by adsorbed HCO_3_^−^ as well as Ca^2+^, Mg^2+^. Moreover, the extent of desorption was greater than the competitive effect of HCO_3_^−^ alone [72]. In terms of the saturation index (SI) of the minerals (Figure 6a), such as goethite and hematite were found to be supersaturated, whereas ferrihydrite and carbonate minerals were either saturated or unsaturated, indicating that Ca^2+^ and Mg^2+^ had not yet reached the critical nucleation point. After 7 days of leaching, the carbonate minerals began to dissolve. In addition, Figure 6b,c indicate that the contents of As combined with carbonate and HCO_3_^−^ concentration, as well as As and Ca^2+^ or Mg^2+^ concentration, exhibited a negative correlation, respectively. Ca^2+^ and Mg^2+^ inhibited the desorption process by increasing the positive charge density on the surface of amorphous Fe oxides and by facilitating electrostatic interactions of As on the surface, which may enhance As adsorption [73]. Moreover, dissolved As in porewater samples was also subjected to competitive adsorption by PO_4_^3−^ (Figure 6d). Due to the similar chemical structure of phosphorus and As element, PO_4_^3−^ formed inner-sphere complexes in Fe oxide surface [74,75], which reduced the binding strength of As to the Fe oxide surface. Thus, it affects the stability of Fe-As complexes. In addition, PO_4_^3−^ directly or indirectly affected the reductive dissolution of Fe oxides and the mineralization pathway of Fe oxides through microbial action, which alters the associated mobilization of As in soil [76,77,78,79]. Furthermore, As concentration had a significant negative correlation with SO_4_^2−^. Studies have reported that SO_4_^2−^ inhibited the transfer of solid-phase As into the liquid phase, which may be related to microbially mediated SO_4_^2−^ reduction [80,81,82].

#### 3.3.3. The Main Controlling Factors for As Migration

**Figure 7:**
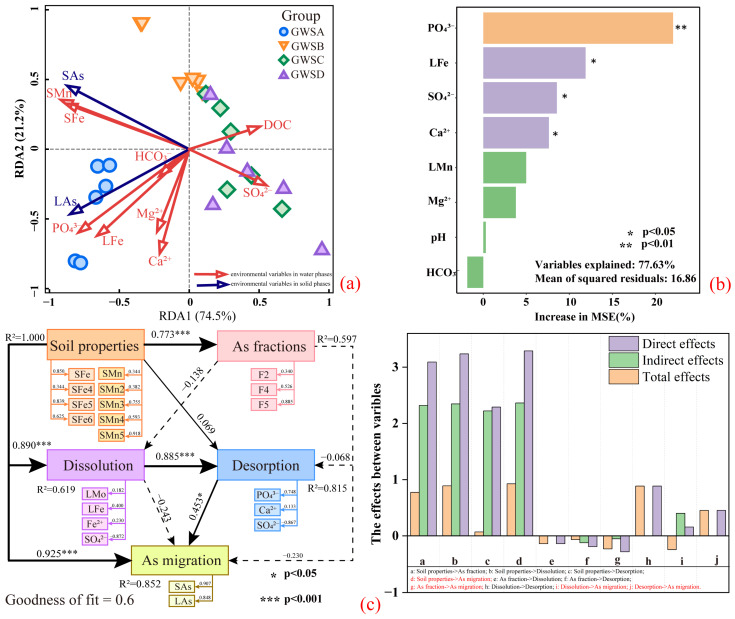
(**a**) Redundancy analysis (RDA); (**b**) Random forest model analysis (RFM); (**c**) Partial least squares path modeling analysis (PLS-PM).

RDA and random forest model analysis were conducted to explore the main controlling factors affecting As migration in soil, while PLS-PM was used to quantitatively evaluate the direct and indirect effects of multiple variables on As migration. According to the RDA analysis (Figure 7a), the parameters explained the variation of total As concentration to a certain extent, with RDA1 and RDA2 accounting for 74.5% and 21.2%, respectively. One group consisted of samples from layer #A (>10 μg/L), which showed a positive correlation between total As concentration and the concentrations of Fe and PO_4_^3−^ concentrations; the other group was the samples for layer #B, C, D (<10 μg/L), which were related to factors (SO_4_^2−^). Further, the random forest model was used to predict the extent of the influence of relevant parameters on the As concentration in porewater. The results (Figure 7b) showed that each variable explained about 77.63% of the total variance, with PO_4_^3−^, total Fe, SO_4_^2−^, and Ca^2+^ concentrations having significant effects on the total As concentration. The order of influence for the environmental variable parameters was as follows: PO_4_^3−^ (InMSE% = 22.04) > Fe (InMSE% = 11.88) > SO_4_^2−^ (InMSE% = 8.54) > Ca^2+^ (InMSE% = 7.61). Both Fe and S are redox-sensitive elements, likely related to the redox environment and microbially mediated redox reactions [83,84]. This indirectly suggests that redox reactions lead to the dissolution of Fe oxides, which contain As and are one of the main driving factors for As migration. PO_4_^3−^, SO_4_^2−^ and Ca^2+^ affect As migration in soil through adsorption and desorption processes. PLS-PM (Figure 7c) demonstrates a good fit for the data (Goodness of fit = 0.60). Soil properties (solid Fe/Mn fraction) directly affected the solid As fraction (r = 0.773) and the reductive dissolution of minerals (r = 0.890). The dissolution process had a direct positive effect on the desorption process (r = 0.885), which in turn positively affected As migration in soil (r = 0.453). According to the sequencing results (Figure S11), the functional genes of As transformation were found to be dominated by reducing genes (*arsC*), which were primarily present in *Sphingomonas*_sp., *Microvirga_ossetica*, *Acidobacteriota_bacterium*. Therefore, the reduction of As(V) to As (III) may have resulted in the desorption of As from the surface of Fe oxides. The results indicate that soil properties (solid Fe/Mn fraction) are the main controlling factors for As migration in soil (r = 0.925).

### 3.4. The Mechanisms of As Migration in Cultivated Soils

**Figure 8:**
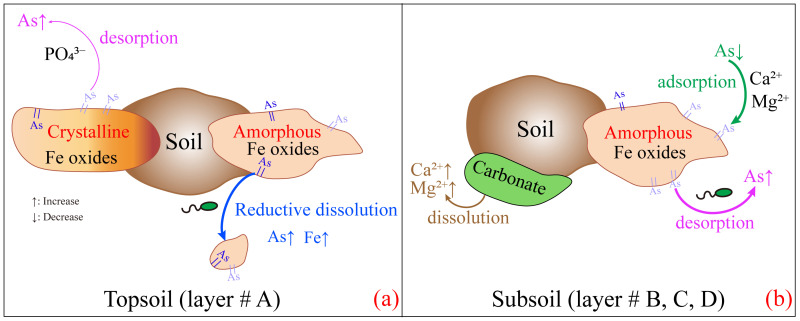
As migration mechanism in soil irrigated by the contained As brackish groundwater. (**a**,**b**) represent the mechanisms of arsenic migration in the Topsoil and subsoil, respectively.

In summary, the As migration mechanism in the soil leaching process is depicted in Figure 8. The reductive dissolution and desorption processes of As from the amorphous Fe oxides were the main sources of As for soil pore water in the topsoil (layer #A) and subsoil (layers #B, C, D), respectively. The presence of oxyanions (PO_4_^3−^, SO_4_^2−^) influenced the transfer of solid-phase As into the liquid phase. In the topsoil (Figure 8a), PO_4_^3−^ was a relatively significant predictor of As concentration in pore water and controlled the process by which As was desorbed from crystalline Fe oxides. More importantly, soil properties gradually changed as irrigation activities occurred. This resulted in the dissolution of Fe/Mn oxides due to redox reactions, which may trigger As desorption from mineral surfaces and ultimately facilitate its migration. In the subsoil (Figure 8b), the increase in Ca^2+^ concentration due to the dissolution of carbonate and gypsum enhanced the electrostatic interactions with dissolved As and inhibited As desorption. Moreover, microbial activities may also play a role in As mobilization during the reductive dissolution of Fe oxides and the desorption process. Therefore, As-contaminated in brackish groundwater irrigation promoted As migration in soil, which correspondingly increased the ecological risk of As pollution in the topsoil.

## 4. Conclusions

The reductive dissolution and desorption processes both played prominent roles in regulating the migration of As in cultivated soil irrigated by brackish groundwater containing As. However, there were significant differences in the predominant driving processes and the main controlling factors across different soil profiles. At the initial stage of leaching, the As concentration in pore water was influenced by the adsorption process in the topsoil (layer #A), while it primarily arose from the desorption of As from amorphous Fe oxides in the subsoil (layers #B, C, D). After 7 days of leaching, the As-loaded amorphous Fe oxides began to undergo reductive dissolution in the topsoil, which promoted competitive adsorption with PO_4_^3−^ in the aqueous phase. In the subsoil, the increase in Ca^2+^ and Mg^2+^ concentrations for the porewater inhibited the desorption of As from amorphous Fe oxides. Meanwhile, the dominant microbial communities were primarily from *Actinomycetota*, *Acidobacteriota,* and *Pseudomonadota* in soils. Therefore, As-contaminated brackish groundwater irrigation promotes As migration in soil and will partly generate ecological risks for cultivated soil.

Taking As-contaminated brackish water and soil as examples, this study enhances the understanding of As fate and evolution in dynamic environments and serves as a reference for future studies in similar scenarios. It contributes to the clarification of the complex geochemical processes affecting As behavior during the interaction between brackish water and soil under dynamic conditions. In the future, we should pay more attention to the microbial driving mechanisms in soil, which will contribute to comprehensively understanding the changes in complex processes under oscillating redox conditions and elemental coupling affecting As behavior in soil. In addition, whether soil leachate increases ecological risks and impacts groundwater quality and safety will need to be validated.

## Data Availability

The original contributions presented in this study are included in the article/Supplementary Materials. Further inquiries can be directed to the corresponding author.

## Supplementary Materials

The following supporting information can be downloaded at: https://www.mdpi.com/article/10.3390/microorganisms12122385/s1, Figure S1: The source of Ca^2+^ and HCO_3_^−^ in soil porewater: (a) the ratio of Ca^2+^ and SO_4_^2−^ concentration; (b) the ratio of Ca^2+^ and HCO_3_^−^ concentration; (c) the ratio of HCO_3_^−^ and (Cl^−^ + SO_4_^2−^) concentration; (d) the ratio of HCO_3_^−^ and DOC concentration. Figure S2: Correlation analysis about the total As concentration (LAs) and environmental factors for porewater. Figure S3: Correlation analysis about the total As/Fe concentration (LAs) and ORP for porewater. Figure S4: The composition of functional microorganisms for As. Figure S5: The As content and standard errors based on different extraction phases for extraction standard (BCR-701). Figure S6: The relationship between the As concentration in porewater and the adsorbed As (F2). Figure S7: Arsenic pe−pH diagram (condition: 25 °C; 1 μmol/L As; 10 μmol/L Fe): (a) As−O_2_−H_2_O system; (b) Fe−As−H_2_O (represented by Hfo adsorbed species). The diagrams use PhreePlot to plot and the the Dzombak & Morel (1990) DL model for Hfo estimate As adsorption by Hfo. Figure S8: (a) Relation between amorphous Fe content and strongly adsorbed As for soil samples; (b) Relation between crystalline Fe content and strongly adsorbed As for soil samples. Figure S9: The change trend of the adsorbed As (F2), amorphous Fe oxides combined As (F4) and crystallized Fe oxides combined As (F5) contents with time for soil samples of layer #A (a), B (b), C (c), D (d). Figure S10: Relation between the As concentration and the contents of solid phase As (carbonates and crystalline Fe oxides bond As). Figure S11: (a) The relative abundance of As functional genes in Sample GWSA-7d. (b) The relative abundance of microorganisms for the gene of *arsC* in Sample GWSA-7d. Table S1: Physicochemical properties of the experimental soil after air-drying and sieving at room temperature. Table S2: Steps of sequential extraction for arsenic and iron. Table S3. Hydrochemical composition of soil porewater in the column experiment.
