# Supplementary material for: Factors and Mechanisms Affecting Arsenic Migration in Cultivated Soils Irrigated with Contained Arsenic Brackish Groundwater
Source: Microorganisms. 2024 Nov 21;12(12):2385. doi: 10.3390/microorganisms12122385 (PMC11677285; doi:10.3390/microorganisms12122385)
Supplement: Supplementary file 1 [file microorganisms-12-02385-s001.zip › microorganisms-3325553-supplementary.pdf]

Supplemental Material

Factors and mechanisms for affecting arsenic migration in cultivated soils irrigated with contained arsenic brackish groundwater

Wen-Jing Dai [a, b], Rong-Guang Shi [c], Xiao-Dong Li [a], Zhi-Qi Zhao [b], Zi-Han Xia [b], Dong-Li Li [a], Yan-Li [a], Gao-Yang Cui [d], Shi-Yuan Ding [a*]

[a] School of Earth System Science, Tianjin University, Tianjin 300072, China

[b] School of Earth Science and Resource, Chang'an University, Xi'an 710054, China

[c] Agro-Environmental Protection Institute, Ministry of Agriculture and Rural Affairs, Tianjin 300072, China

[d] College of Geography and Environmental Science, Henan University; Kaifeng 475004, Henan, China.

* Corresponding author: Shi-Yuan Ding, School of Earth System Science, Tianjin University, Tianjin 300072, China. E-mail address: dingshiyuan@tju.edu.cn

Content:

(1) Figures: S1-S11

Figure S1. The source of Ca^{2+} and HCO_3^- in soil porewater: (a) the ratio of Ca^{2+} and SO_4^{2-} concentration; (b) the ratio of Ca^{2+} and HCO_3^- concentration; (c) the ratio of HCO_3^- and (Cl^-+ SO_4^{2-}) concentration; (d) the ratio of HCO_3^- and DOC concentration.

Figure S2. Correlation analysis about the total As concentration (LAs) and environmental factors for porewater.

Figure S3. Correlation analysis about the total As/Fe concentration (LAs) and ORP for porewater.

Figure S4.The composition of functional microorganisms for As.

Figure S5. The As content and standard errors based on different extraction phases for extraction standard (BCR-701).

Figure S6. The relationship between the As concentration in porewater and the adsorbed As (F2).

Figure S7. Arsenic pe−pH diagram (condition: 25°C; 1 μmol/L As; 10 μmol/L Fe): (a) As−O_2−H_2O system; (b) Fe−As−H_2O (represented by Hfo adsorbed species). The diagrams use PhreePlot to plot and the the Dzombak & Morel (1990) DL model for Hfo estimate As adsorption by Hfo.

Figure S8. (a) Relation between amorphous Fe content and strongly adsorbed As for soil samples; (b) Relation between crystalline Fe content and strongly adsorbed As for soil samples.

Figure S9. The change trend of the adsorbed As (F2), amorphous Fe oxides combined As (F4) and crystallized Fe oxides combined As (F5) contents with time for soil samples of layer #A (a), B (b), C (c), D (d)

Figure S10. Relation between the As concentration and the contents of solid phase As (carbonates and crystalline Fe oxides bond As)

Figure S11. (a)The relative abundance of As functional genes in Sample GWSA-7d. (b) The relative abundance of microorganisms for the gene of *arsC* in Sample GWSA-7d..

Tables: S1-S3

Table S1. Physicochemical properties of the experimental soil after air-drying and sieving at room temperature.

Table S2. Steps of sequential extraction for arsenic and iron

Table S3. Hydrochemical composition of soil porewater in the column experiment.

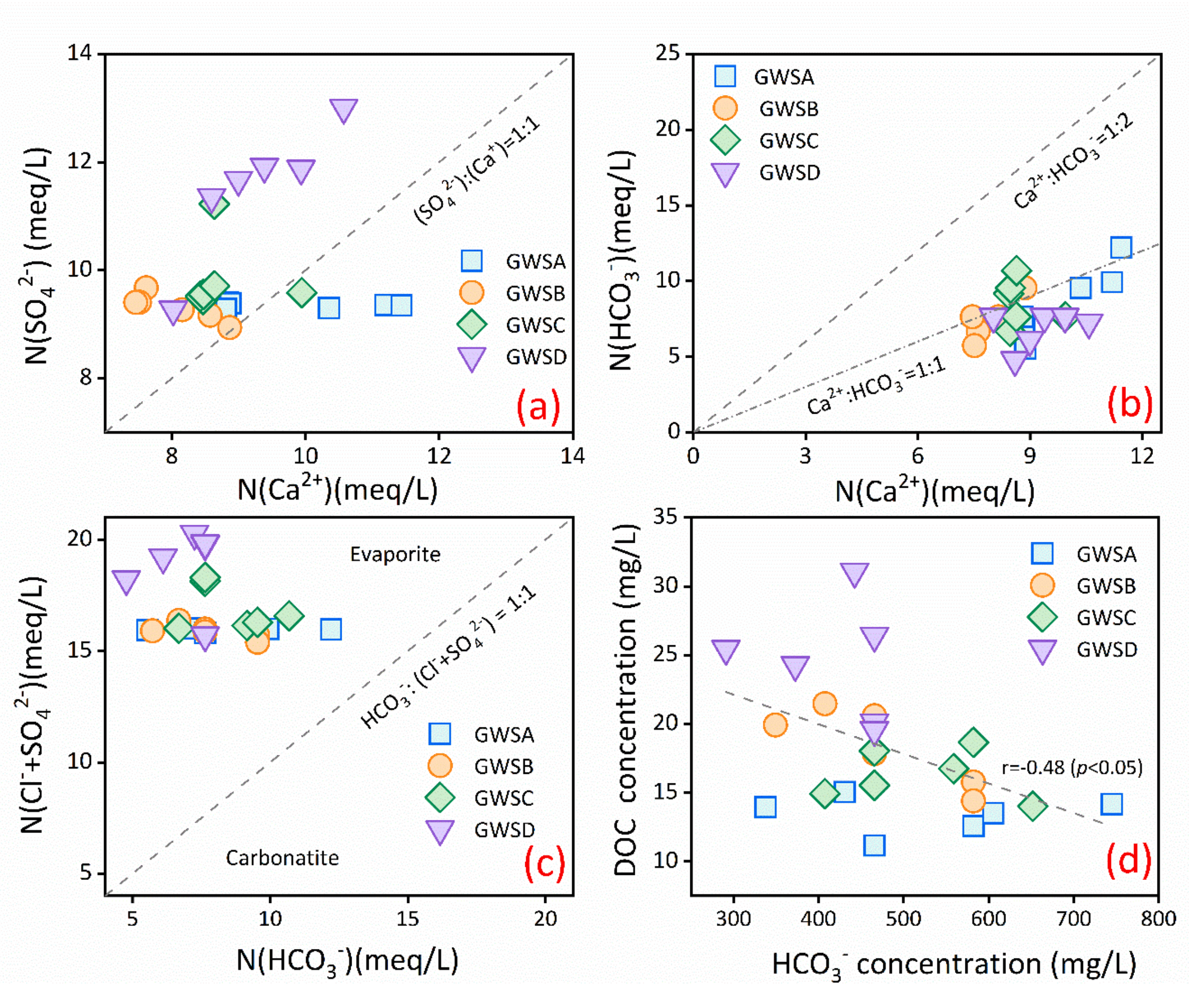

Figure S1. The source of Ca^{2+} and HCO_3^- in soil porewater: (a) the ratio of Ca^{2+} and SO_4^{2-} concentration; (b) the ratio of Ca^{2+} and HCO_3^- concentration; (c) the ratio of HCO_3^- and (Cl^-+ SO_4^{2-}) concentration; (d) the ratio of HCO_3^- and DOC concentration.

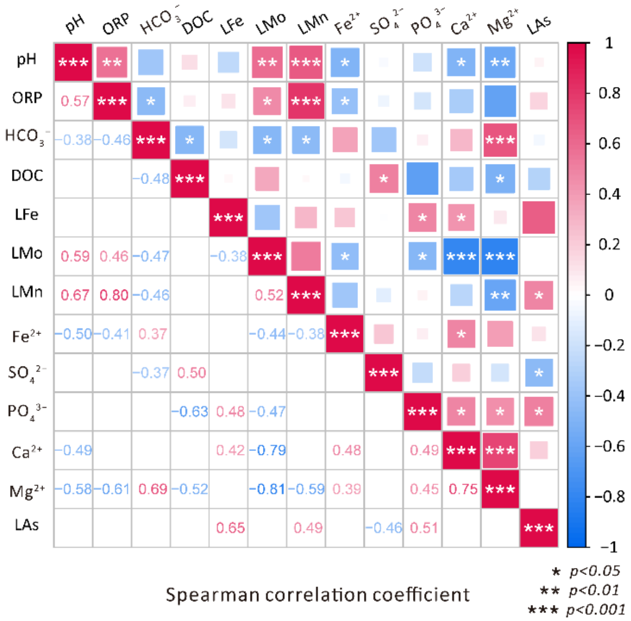

Figure S2. Correlation analysis about the total As concentration (LAs) and environmental factors for porewater.

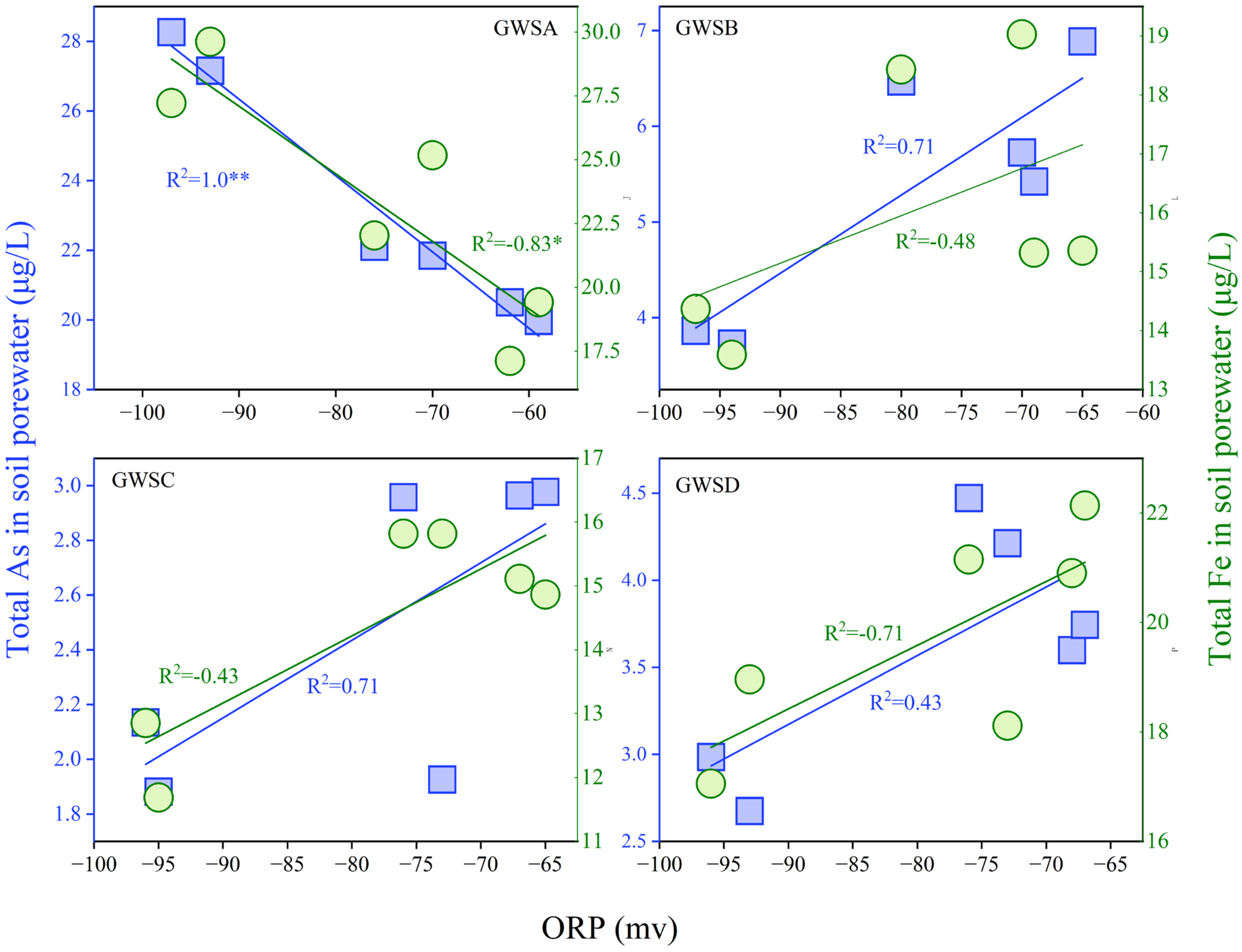

Figure S3. Correlation analysis about the total As/Fe concentration (LAs) and ORP for porewater.

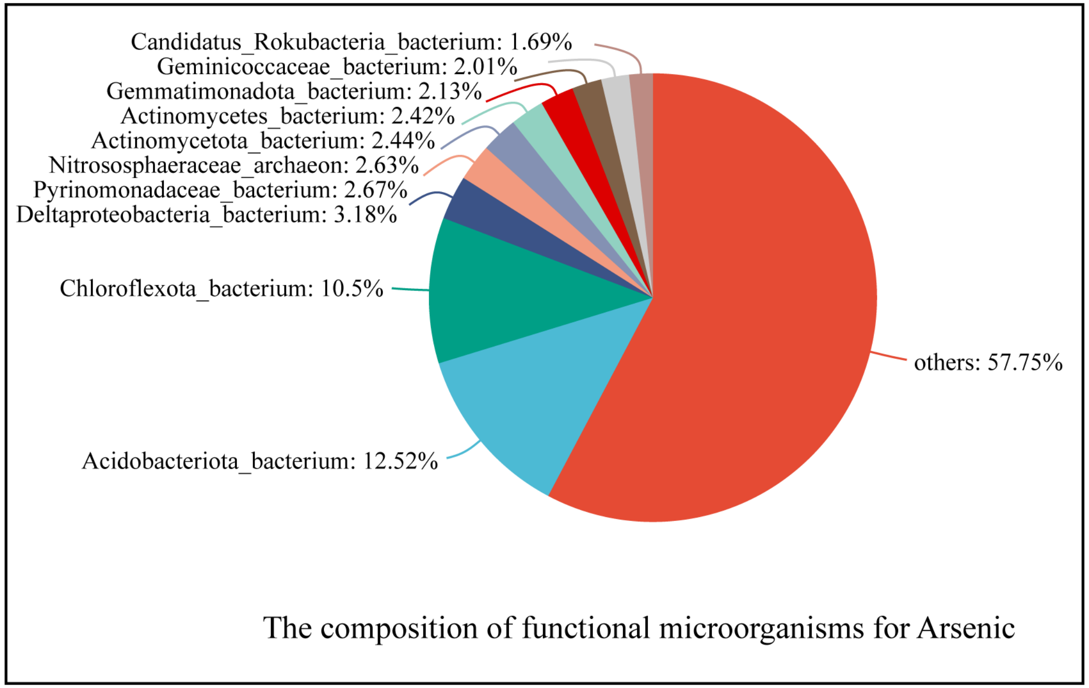

Figure S4.The composition of functional microorganisms for As.

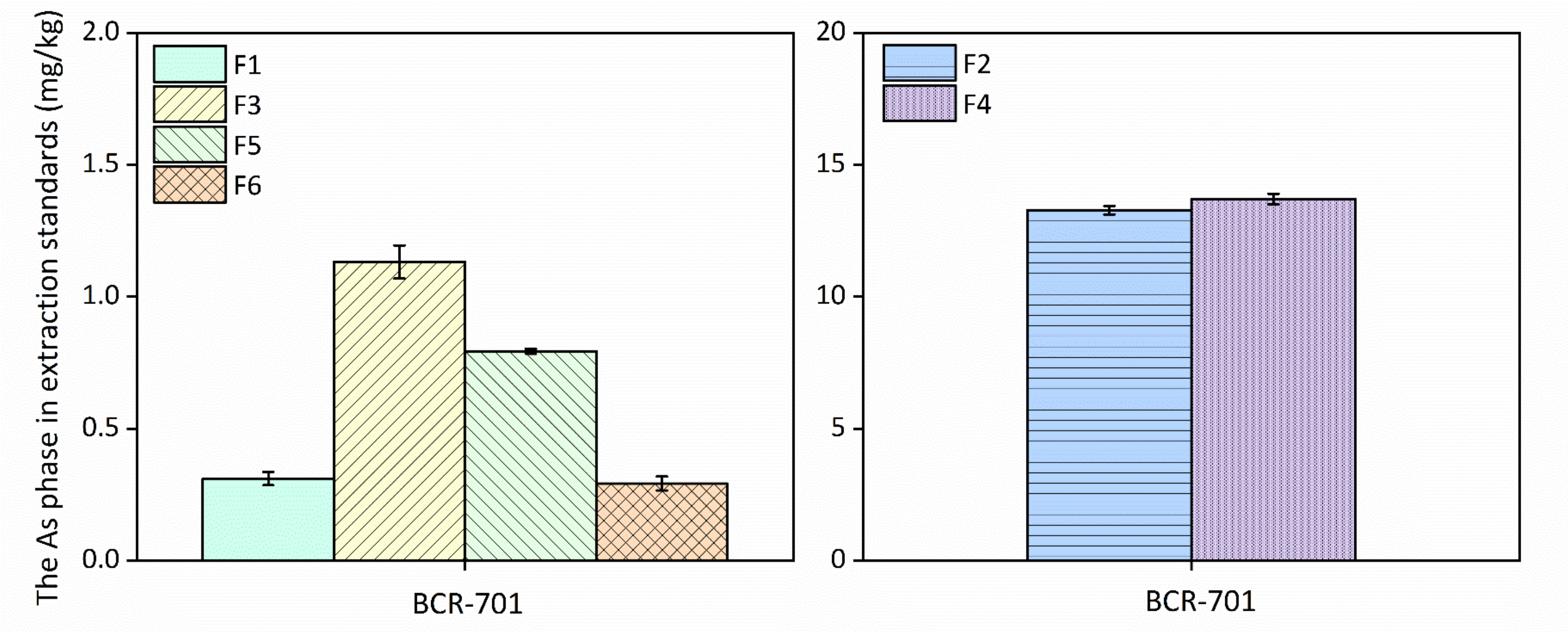

Figure S5. The As content and standard errors based on different extraction phases for extraction standard (BCR-701).

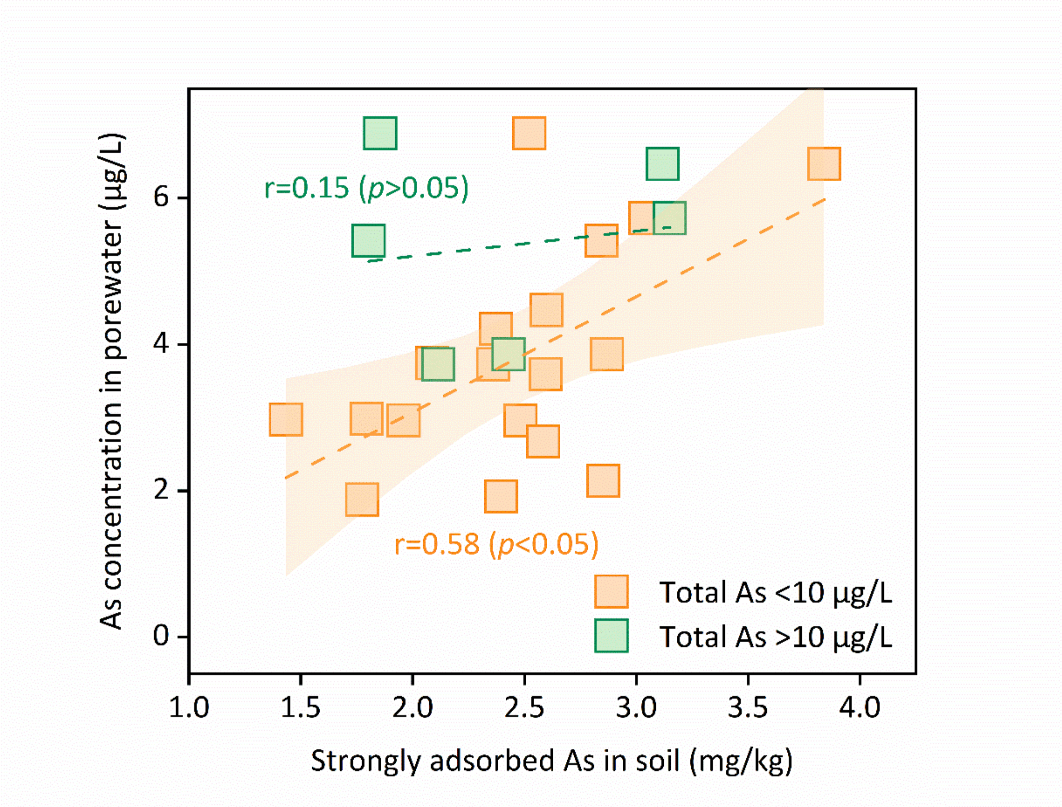

Figure S6. The relationship between the As concentration in porewater and the adsorbed As (F2).

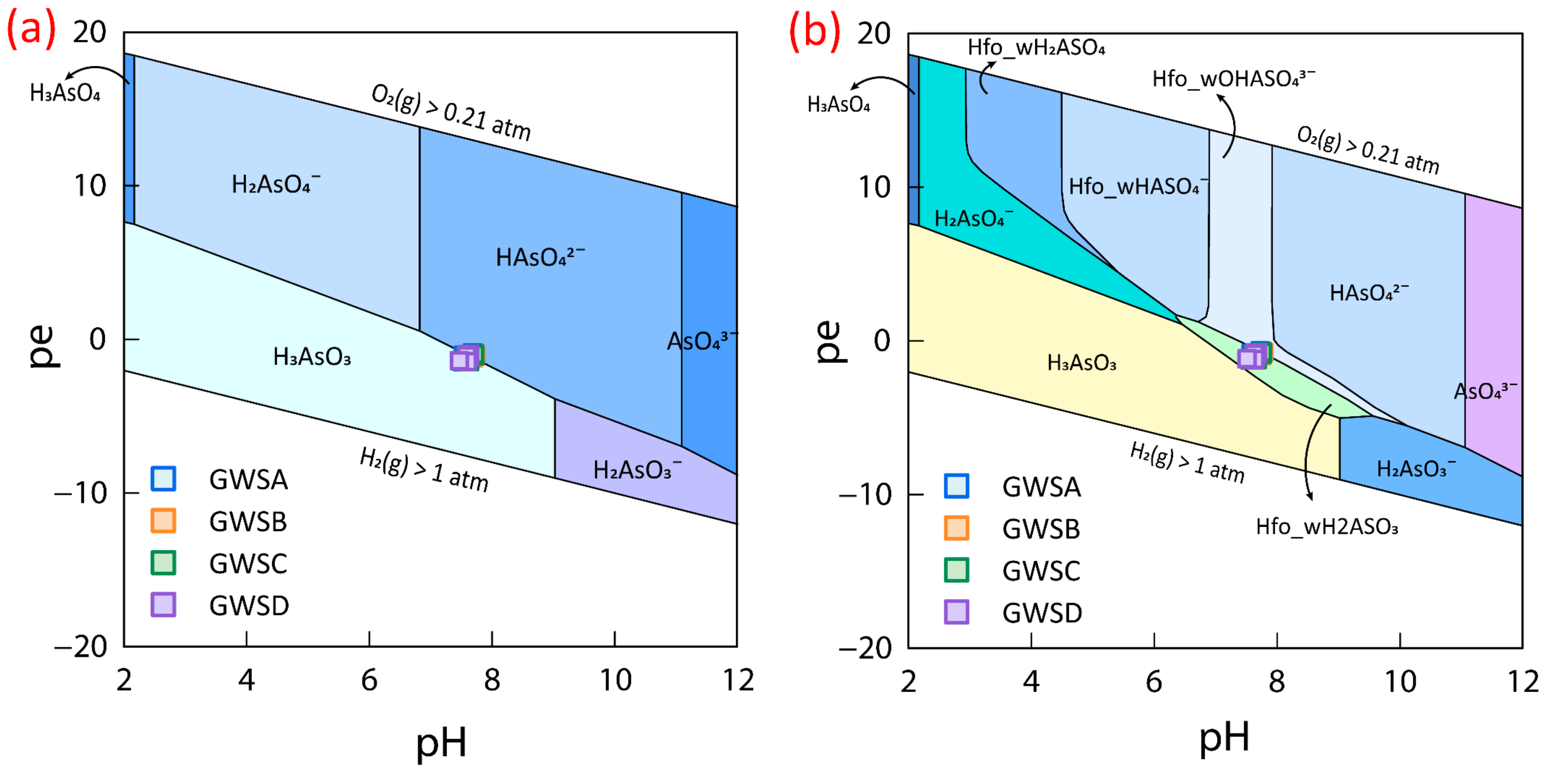

Figure S7. Arsenic pe−pH diagram (condition: 25°C; 1 µmol/L As; 10 µmol/L Fe): (a) As−O_2−H_2O system; (b) Fe−As−H_2O (represented by Hfo adsorbed species). The diagrams use PhreePlot to plot and the the Dzombak & Morel (1990) DL model for Hfo estimate As adsorption by Hfo.

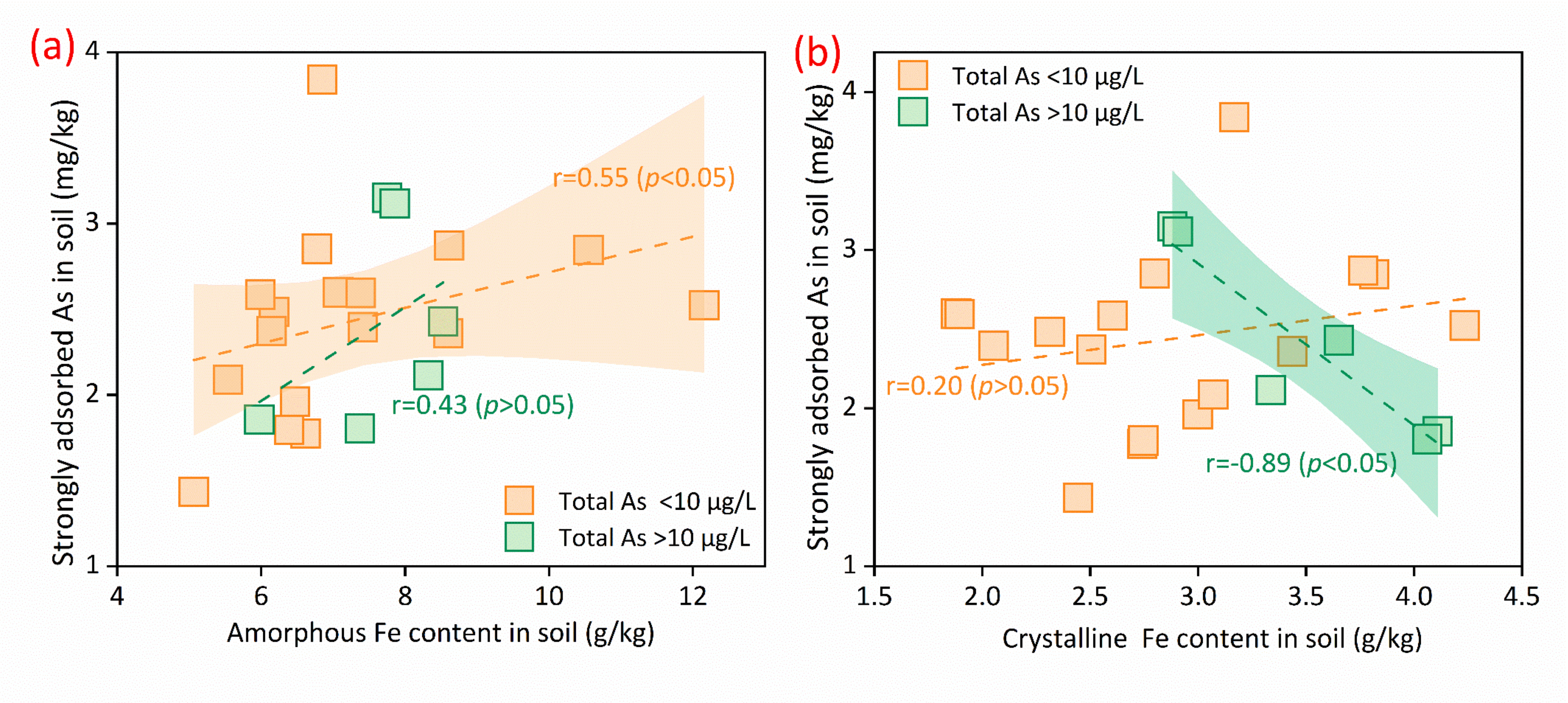

Figure S8. (a) Relation between amorphous Fe content and strongly adsorbed As for soil samples; (b) Relation between crystalline Fe content and strongly adsorbed As for soil samples.

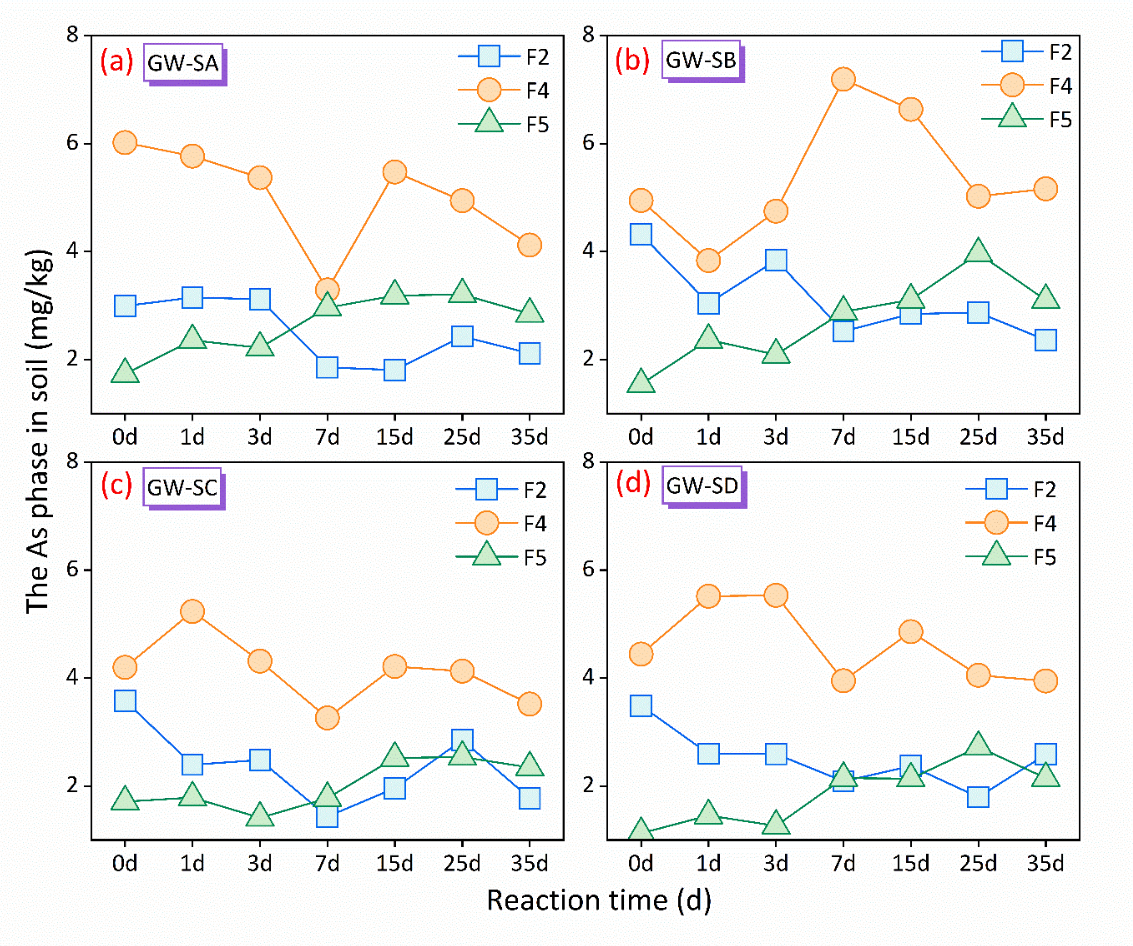

Figure S9. The change trend of the adsorbed As (F2), amorphous Fe oxides combined As (F4) and crystallized Fe oxides combined As (F5) contents with time for soil samples of layer #A (a), B (b), C (c), D (d)

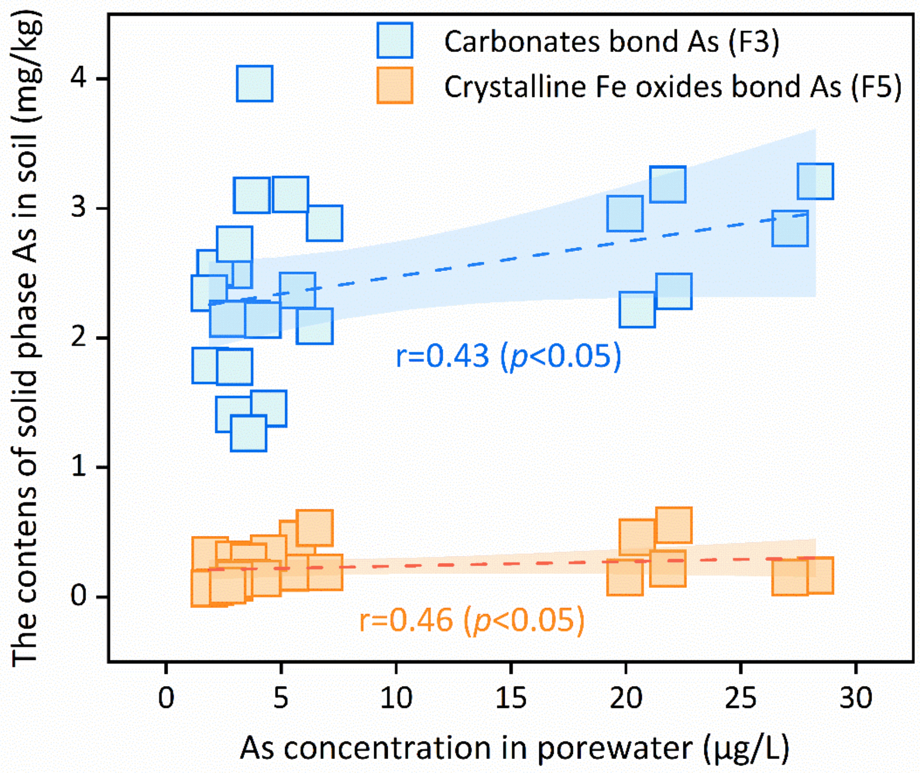

Figure S10. Relation between the As concentration and the contents of solid phase As (carbonates and crystalline Fe oxides bond As)

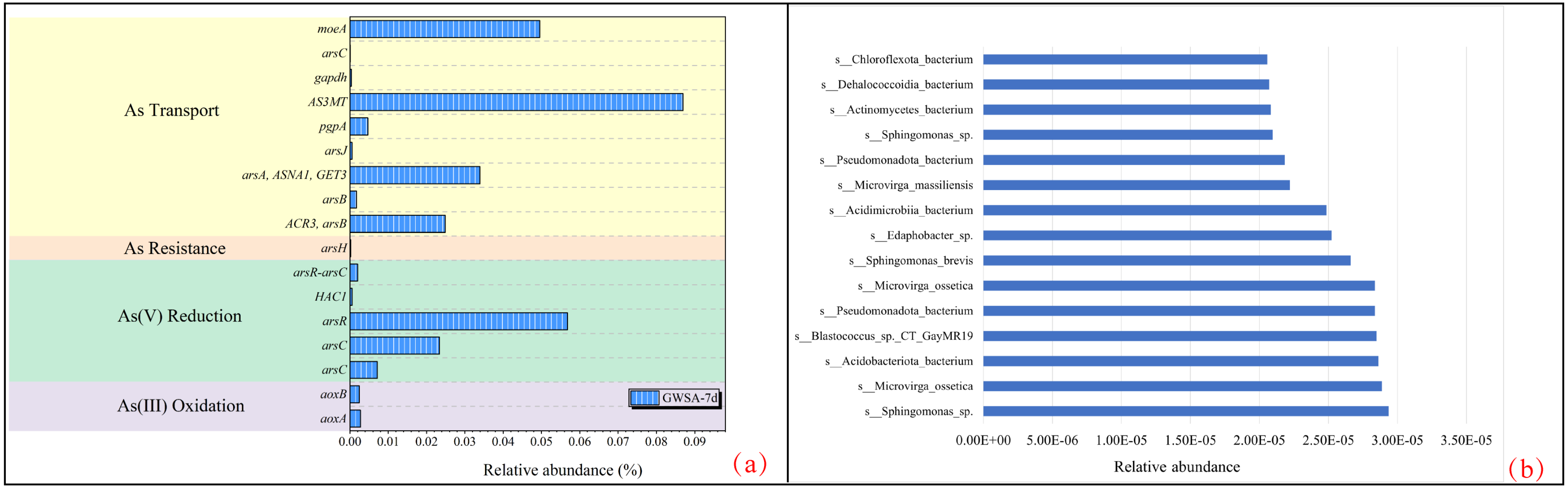

Figure S11. (a)The relative abundance of As functional genes in Sample GWSA-7d. (b) The relative abundance of microorganisms for the gene of *arsC* in Sample GWSA-7d..

Table S1. Physicochemical properties of the experimental soil after air-drying and sieving at room temperature.

Layer	Location [a]	soil bulk density (g/cm^3)	pH	EC (μs/cm)	Organic carbon (wt%)	Total Nitrogen (wt%)	Cation exchange capacity (CEC, cmol$^+$/kg)	As (mg/kg)	Fe (g/kg)	Mn (mg/kg)
GWSA	0~35	1.778	8.08 ± 0.06	297 ± 8	2.46	0.119	59.0 ± 0.4	13.8	30.1	721.7
GWSB	35~60	1.790	8.16 ± 0.05	244 ± 7	1.12	0.051	61.2 ± 0.1	14.8	31.1	666.5
GWSC	60~80	1.500	8.35 ± 0.14	198 ± 34	0.42	0.020	66.5 ± 0.1	9.00	19.0	449.0
GWSD	80~120	1.629	8.33 ± 0.12	179 ± 25	0.49	0.021	67.6 ± 0.1	10.0	20.3	461.2
Local soil background value (topsoil)								6.1	33	570.6
Local soil background value (subsoil)								8.0	33	501.5

[a]: The depth from the ground surface (cm)

Table S2. Steps of sequential extraction for arsenic and iron

Step	extractant	extraction condition	defined	As fraction	Fe fraction	Mn fraction
1	1 mol/L magnesium chloride, pH=7.0±0.2	25°C, 24h in dark	Non-specific adsorbed	F1	–	–
2	1 mol/L sodium dihydrogen phosphate, pH=5.0±0.2 [a]	25°C, 24h in dark	Strongly adsorbed	F2	–	SMn2
3	1mol/L acetic acid sodium, pH=4.5 [a]	25°C, 24h in dark	Carbonates bound	F3	SFe3	SMn3
4	1 mol/L hydroxylamine−hydrochloride in 25% v/v acetic acid [a]	25°C, 48h in dark	Amorphous Fe oxides (Ferrihydrite and lepidocrocite) associated	F4	SFe4	SMn4
5	50 g/L sodium dithionite, pH=4.8 (buffered solution: 0.35 mol/L acetic acid/0.2 mol/L sodium citrate) [a]	25°C, 2 h in dark	Crystalline Fe oxides (Goethite and hematite) associated	F5	SFe5	SMn5
6	0.2mol/L ammonium oxalate and 0.17 mol/L oxalic acid, pH=3.2 [a]	25°C,6.5 h, 2 h in dark	Recalcitrant Fe oxides (Magnetite) associated	F6	SFe6	SMn6−

[a]: Samples were washed with Milliq water between two different extractant for 30 min in thermostatic oscillator. Then a centrifuge with 4000 rpm/min for 10min separated the solid and the liquid.

Table S3. Hydrochemical composition of soil porewater in the column experiment.

Sample ID	pH	EC (ms/cm)	ORP (mV)	Na^{+} (mg/L)	K^{+} (mg/L)	Mg^{2+} (mg/L)	Ca^{2+} (mg/L)	Cl^{-} (mg/L)	HCO_3^{-} (mg/L)	SO_4^{2-} (mg/L)	NO_2^{-} (mg/L)	SiO_3^{2-} (mg/L)	PO_4^{3-} (mg/L)	As (μg/L)	Fe (μg/L)
GWSA−1d	7.77	1.84	−76	145	9.98	71.6	177	235	431	452	0.252	8.10	0.630	22.1	22.0
GWSA−3d	7.85	1.82	−62	142	9.22	72.5	178	233	338	451	0.102	7.41	0.660	20.5	17.1
GWSA−7d	7.79	1.64	−59	140	8.63	73.6	176	232	466	446	0.122	6.26	0.575	20.0	19.4
GWSA−15d	7.55	1.92	−70	150	8.66	84.9	207	235	582	446	0.287	8.23	0.693	21.8	25.2
GWSA−25d	7.52	2.50	−97	153	8.19	96.7	224	236	605	449	0.082	8.37	0.859	28.3	27.2
GWSA−35d	7.48	2.63	−93	160	9.12	111	229	235	745	449	0.322	10.37	0.889	27.2	29.6
GWSB−1d	7.72	1.93	−70	214	12.16	63.5	152	238	407	464	0.122	5.38	0.060	5.73	19.0
GWSB−3d	7.82	1.87	−80	202	10.45	63.9	150	231	349	452	0.452	4.79	0.080	6.47	18.4
GWSB−7d	7.88	1.65	−65	193	9.75	64.1	149	234	466	451	0.372	3.73	0.030	6.88	15.4
GWSB−15d	7.75	1.56	−69	193	11.1	69.8	163	234	466	445	0.052	4.84	0.020	5.42	15.3
GWSB−25d	7.67	1.77	−97	190	10.2	79.7	171	233	582	440	0.362	4.98	0.055	3.86	14.4
GWSB−35d	7.54	1.92	−94	193	11.2	90.4	177	229	582	429	0.092	6.91	0.066	3.73	13.6
GWSC−1d	7.85	2.30	−73	216	2.55	81.0	199	304	466	460	0.172	3.95	0.070	1.93	15.8
GWSC−3d	7.61	2.22	−76	206	3.25	70.7	169	233	407	454	0.092	4.34	0.300	2.96	15.8
GWSC−7d	7.84	1.88	−65	237	3.09	63.9	173	251	466	539	0.292	3.38	0.020	2.98	14.9
GWSC−15d	7.69	2.30	−67	203	2.82	70.6	168	235	559	457	0.047	3.93	0.019	2.96	15.1
GWSC−25d	7.71	1.74	−96	203	2.47	76.9	169	239	582	459	0.262	4.28	0.045	2.13	12.9
GWSC−35d	7.7	1.76	−95	213	2.90	85.3	173	244	652	466	0.112	5.60	0.061	1.88	11.7
GWSD−1d	7.7	2.49	−76	259	9.49	79.2	211	258	442	624	5.79	0.48	0.060	4.47	21.1
GWSD−3d	7.65	1.97	−68	235	3.09	65.2	172	244	291	545	2.00	3.98	0.290	3.60	20.9
GWSD−7d	7.72	2.27	−67	193	2.23	66.2	160	228	466	445	0.172	3.76	0.010	3.74	22.1
GWSD−15d	7.63	2.28	−73	247	4.36	68.1	180	267	373	560	0.047	4.26	0.018	4.21	18.1
GWSD−25d	7.65	1.91	−96	252	2.98	76.3	188	279	466	572	0.092	4.21	0.051	2.98	17.1
GWSD−35d	7.44	2.94	−93	261	3.13	87.8	199	283	466	570	0.072	6.28	0.047	2.67	19.0
Groundwater	−	2.0	−	141	7.2	61.3	182	230	−	413	−	6.73	0.010	8.05	18.9
